# Short-term MRI measurements as predictors of EDSS progression in relapsing-remitting multiple sclerosis: grey matter atrophy but not lesions are predictive in a real-life setting

**DOI:** 10.7717/peerj.2442

**Published:** 2016-09-20

**Authors:** Johanna von Gumberz, Mina Mahmoudi, Kim Young, Sven Schippling, Roland Martin, Christoph Heesen, Susanne Siemonsen, Jan-Patrick Stellmann

**Affiliations:** 1 Institute of Neuroimmunology and MS (INIMS), University Medical Center Hamburg-Eppendorf, Hamburg, Germany; 2 Department of Neurology, University Medical Center Hamburg-Eppendorf, Hamburg, Germany; 3 Neuroimmunology and Multiple Sclerosis Research Section, Department of Neurology, University Hospital Zürich, Zürich, Switzerland; 4 Department of Diagnostic and Interventional Neuroradiology, University Medical Center Hamburg-Eppendorf, Hamburg, Germany

**Keywords:** Multiple sclerosis, MRI, Disability progression, Predictors, Atrophy, Lesions

## Abstract

**Background:**

Magnetic resonance imaging (MRI) is the best biomarker of inflammatory disease activity in relapsing remitting Multiple Sclerosis (RRMS) so far but the association with disability is weak. Appearance of new MRI-lesions is used to evaluate response to immunotherapies in individual patients as well as being the most common primary outcome in phase-2 trials. Measurements of brain atrophy show promising outcomes in natural cohort studies and some phase-2 trials. From a theoretical perspective they might represent irreversible neurodegeneration and be more closely associated with disability. However, these atrophy measurements are not yet established as prognostic factors in real-life clinical routine. High field MRI has improved image quality and resolution and new methods to measure atrophy dynamics have become available.

**Objective:**

To investigate the predictive value of MRI classification criteria in to high/low atrophy and inflammation groups, and to explore predictive capacity of two consecutive routine MRI scans for disability progression in RRMS in a real-life prospective cohort.

**Methods:**

82 RRMS-patients (40 untreated, 42 treated with immunotherapies, mean age 40 years, median Expanded Disability Status Scale (EDSS) of 2, underwent two clinically indicated MRI scans (3 Tesla) within 5–14 months, and EDSS assessment after a mean of 3.0 (1.5–4.2) years. We investigated the predictive value of predefined classifications in low/high inflammatory and atrophy groups for EDSS progression (≥1.5 if baseline EDSS = 0, ≥1.0 if baseline EDSS <5, ≥0.5 for other) by chi-square tests and by analysis of variance (ANOVA). The classifications were based on current scientific or clinical recommendation (e.g., treatment response criteria). Brain atrophy was assessed with three different methods (SIENA, SIENAX, and FreeSurfer). Post-hoc analyses aimed to explore clinical data and dynamics of MRI outcomes as predictors in multivariate linear and logit models.

**Results:**

Progression was observed in 24% of patients and was independent from treatment status. None of the predefined classifications were predictive for progression. Explorative post-hoc analyses found lower baseline EDSS and higher grey matter atrophy (FreeSurfer) as best predictors (*R*^2^ = 0.29) for EDSS progression and the accuracy was overall good (Area under the curve = 0.81).

**Conclusion:**

Beside EDSS at baseline, short-term grey matter atrophy is predictive for EDSS progression in treated and untreated RRMS. The development of atrophy measurements for individual risk counselling and evaluation of treatment response seems possible, but needs further validation in larger cohorts. MRI-atrophy estimates from the FreeSurfer toolbox seem to be more reliable than older methods.

## Introduction

Disability progression in Multiple Sclerosis (MS) is mediated by acute inflammation as well as chronic inflammation and neurodegeneration (Hauser, Chan & Oksenberg, 2013; Friese, Schattling & Fugger, 2014). Magnetic Resonance Imaging (MRI) is currently the best available biomarker in relapsing-remitting Multiple Sclerosis (RRMS) (Hauser, Chan & Oksenberg, 2013) and new T2-hyperintense or contrast enhancing lesions are outcomes of inflammation in clinical trials (Sormani et al., 2009; Stellmann et al., 2015). New lesions are associated as well with treatment failure in individual patients (Rio et al., 2008). Lesion load at the time of diagnosis and its increase within the first five years are prognostic factors for long-term disability at a group level (Fisniku et al., 2008; Popescu et al., 2013; Tintore et al., 2015). However, the association between clinical and MRI measurements of inflammation and disability progression is moderate at best. In contrast, there is growing evidence that atrophy might be closer associated with disability than lesions (Rocca et al., 2013; Jacobsen et al., 2014). Over 10 years, confirmed disability progression was associated with whole brain atrophy, cortical atrophy and ventricular volume (Zivadinov et al., 2016). Cross-sectional studies indicate a better correlation of atrophy with disability and cognitive decline than lesions alone (Benedict, Carone & Bakshi, 2004; Steenwijk et al., 2016). Moreover, atrophy is discussed as an additional criterion to define treatment response within the concept of NEDA (“No evidence of disease activity”) as first studies report on the predictive value of for example percentage brain volume change for treatment response to interferon-beta (Perez-Miralles et al., 2015). However MRI atrophy measurements are not yet established as individual prognostic factors and reliability has not yet been proven in a real-life setting. Furthermore data about short-term atrophy dynamics (e.g., within one year) as predictor of disability progression are rare (Popescu et al., 2013).

Bielekova et al. (2005) were the first to combine simple MRI measurements of inflammation and atrophy as prognostic factors. They aimed to assign patients into four risk groups based on their baseline inflammatory activity (high or low) and respective atrophy (high or low). After eight years the algorithm failed to predict progression. Since then though, high field MRI has improved image quality and resolution and new methods to measure brain atrophy dynamics have become available (Smith et al., 2002; Fischl, 2012). It is therefore reasonable to investigate the predictive value of short-term atrophy and inflammation measurements of two MRI scans in a real life setting, as most patients likely receive them due to clinical monitoring anyway (Uher et al., 2015).

The current study was designed to validate the concept of Bielekova with different classification algorithms representing widely accepted criteria such as the Rio criteria for treatment failure. In addition we aimed to explore as to how far varying atrophy measurements (SIENA/SIENAX from the Functional MRI software library, fmrib.ox.ac.uk and FreeSurfer freesurfer.net) differ in their ability to predict EDSS progression.

## Methods

### Study design

The study was designed to assess the predictive value of two standard MRI scans for EDSS progression in treated and untreated RRMS in a real-life setting. Participants were consecutively recruited and underwent two baseline visits five to 14 months apart including a neurological assessment as well as MRI scans. We scheduled annual follow-up visits but due to an increasing dropout rate (25% in 2014) and a poor compliance to scheduled visits, the study had to be terminated early with final visits in 2014/2015. As a result, patients had heterogeneous follow-up times (median 2.9 years, range 1.5–4.2).

Our analysis plan included two steps: in an hypothesis driven approach, we used short-term changes of lesions and atrophy to define four risk groups and validate their predictive capacities: (I) Low inflammation and low atrophy, (II) high inflammation and low atrophy, (III) low inflammation and high atrophy and (IV) high inflammation and high atrophy. Since the original publication of Bielekova (Bielekova et al., 2005) new methods to measure brain atrophy dynamics became available (Smith et al., 2002; Fischl, 2012). We aimed for a comparison of three frequently used techniques (SIENA, SIENAX, FreeSurfer). Post-hoc, we explored clinical data and different volumetric methods in their ability to predict EDSS progression.

### Patients

Patients aged between 18 and 60 years with a confirmed diagnosis of RRMS according to the revised McDonald Criteria (Polman et al., 2011) had to give written informed consent. Patients were asked to participate at baseline if two MRI scans were clinically indicated within one year. The local ethics committee (Board of Physicians, Hamburg, No. PV4405) approved the study. Between the two baseline visits, patients had to be stable without (untreated) or stable on any disease-modifying drug (DMD, treated). MRI scans were not performed within 30 days after a steroid treatment. 109 patients were enrolled. 56 had a DMD and 53 opted against any DMD in a shared decision process. The expanded disability status scale (EDSS) (Kurtzke, 1983) of all patients was assessed by trained neurologists. Treatment at follow-up was labelled as “no change” or “change”. The kind of treatment change was defined as “escalation”, “no change” and “de-escalation.”

### MRI and image analysis

MRI data were acquired on a 3T scanner including a magnetization prepared rapid acquisition gradient-echo (MPRAGE) T1-weighted sequence (T1, pre-post Gadolinium(Gd)) and a PD-T2-weighted sequence (T2). The software JIM was used to semi-automatically mask lesions in T2 (T2-hyperintense lesions), T1 (T1-hypointense lesions) and T1Gd sequence. Two raters counted lesions and evaluated new lesions. Regions of Interest (ROI) were semi-automatically placed around single lesions in the PD/T2, T1 and T1Gd sequence. The number of lesions was determined manually while volumes were calculated automatically with the ROI-analysis function. Two raters evaluated the number of new T2/BH lesions. Afterwards, all images were processed with the FSL-toolbox (Smith et al., 2004). Brain tissue volume, normalized for subject head size (NBV = normalized brain volume, NGM = normalized grey matter, NWM = normalized white matter), was estimated with SIENAX (Smith et al., 2002) and lesion volume was normalized based on the SIENAX results. To reduce the risk of false tissue assignment in lesions, lesion masks were dilated and filled with normal appearing white matter contrast. Brain masks were manually corrected to minimize false tissue assignment by the FSL-segmentation. Longitudinal atrophy was assessed with SIENA (Smith et al., 2002) and results were corrected for the individual duration between the two baseline scans to calculate an annualized Percentage Brain Volume Change (aPBVC). In addition, we used FreeSurfer (Version 5.2.0, http://surfer.nmr.mgh.harvard.edu/). To extract reliable and comparable volume estimates from both baseline MRI scans, images were processed with the FreeSurfer longitudinal stream (Reuter et al., 2012). We extracted volumes of the grey and white matter. Brain masks and white/grey matter segmentation were also manually corrected if needed.

### Statistics

We performed descriptive statistics according to the nature of the data by means with standard deviation (sd) or as frequencies and/or percentages. Based on a single EDSS at follow-up and the lowest baseline EDSS we calculated absolute change and EDSS progression of each of the patients. Progression was stated if the EDSS increased by 1.5 points or more (baseline EDSS = 0), if the EDSS increased by one or more points (baseline EDSS between one and four) or if the EDSS increased 0.5 points or more (baseline EDSS above five) (Sormani & De Stefano, 2013). All changes were annualized based on the interval between the two baseline visits (i.e., scan one and two). To identify potential cofounders for EDSS progression, we checked if the variable baseline or follow-up time differed between patients with or without EDSS progression (*T*-test). We investigated if baseline variables or follow-up times differ between treated and untreated patients. In case of significant differences we adjusted further analyses for treatment status if possible.

### Predefined criteria

Classification into low and high inflammation was defined by four different criteria:

(A) No lesion vs. at least one lesion per year (representing no inflammatory activity versus any activity, used with the No evidence for disease activity (NEDA) outcome in clinical trials).
(B) Two lesions per year vs. less (MRI-criterion of treatment non-response (Rio et al., 2008)).
(C) Four lesions per year vs. less (extending criteria A/B towards a higher inflammatory cut-off).
(D) One lesion per month (representing the original Bielekova criterion (Bielekova et al., 2005)).

The four corresponding definitions for low and high atrophy groups represented three commonly used methods to assess atrophy:

(1) Absolute change of NBV (any atrophy vs. none atrophy, SIENAX).
(2) SIENA-aPBVC (any atrophy vs. none atrophy).
(3) Total brain volume change from FreeSurfer longitudinal stream (any atrophy vs. none atrophy).
(4) Median split of the absolute NBV of the first MRI (SIENAX, Bielekova et al., 2005).

Median NBV split values were 1,539,505 mm^3^ in untreated and 1,875,934 mm^3^ in treated patients. The predictive value of each combination of criteria (e.g., 1A, 2C, 3B etc.) for disease progression was evaluated by chi-square tests and by analysis of variance (ANOVA).

### Post-hoc exploratory analyses

First, we investigated the ability of the following variables to predict the EDSS change and progression in linear and logit models adjusted for treatment status: gender, age, number of T1-, T2- and Gd-lesions, the absolute change of lesion numbers and SIENAX volumes from Visit 1 to Visit 2, aPBVC; as well as global atrophy measurements from the longitudinal FreeSurfer processing (volumes: brain, white matter, grey matter, subcortical grey matter, cortical grey matter, supratentorial brain). Potential interactions with treatment status were investigated the same way. *P*-values were corrected for multiple testing with the false discovery rate (FDR) method. Remaining significant predictors were afterwards combined in multivariate models by forward stepwise selection of variables based on the Akaike Information Criterion (AIC). To quantify the predictive value of the models we calculated the coefficient of determination (*R*^2^) for linear models. In addition we computed Receiver Operating Characteristic (ROC) curves and their Area under the curve (AUC) from logit models with progression (“yes”, “no”) as a binary outcome. Sensitivity, specificity, and the negative (NPV) and positive predictive value (PPV) were estimated to be at the best threshold from predicted values. Finally, we calculated odds ratios and their 95% Confident Intervals (95% CI) for each variable. All analyses were performed with Statistics in R 3.1.2.

**Table 1:** Descriptive statistics.

	All	Untreate	Treate	*p*-value
	*N* = 82	*N* = 42	*N* = 40	
**Baseline**				
Gender female *n* (%)	53(0.65)	27(0.64)	26(0.65)	1.0^a^
Age year	40.6(9.6)	42.9(9.2)	38.2(9.5)	0.029^b^
Disease duration	7.5(7.7)	6.9(9.3)	8.1(5.5)	0.5^b^
EDSS	1.6(1.4)	1.1(1.2)	2.1(1.4)	<0.001^b^
EDSS median (range)	2 (0–6)	1 (0–5)	2 (0–6)	<0.001^c^
Difference between two MRI month	7.5(2.1)	7.8(2.2)	7.1(1.9)	0.1^b^
T2-lesions n	61(46.6)	50.6(35.9)	71.9(54)	0.040^b^
T1-lesions n	5.9(7.8)	3.2(5.1)	8.8(9.2)	0.001^b^
GD-lesions n	0.2(0.4)	0.2(0.5)	0.1(0.3)	0.036^b^
Delta T2-lesions *n*/year	3(5.7)	3.9(6)	2(5.4)	0.1^b^
Delta T1-lesions *n*/year	0.8(1.5)	0.8(1.5)	0.8(1.5)	1.0^b^
NBV mm^3^	1,673,305(199,072)	1,538,369(85,524)	1,81,4987(185,529)	<0.001^b^
NWM mm^3^	740,634(71,743)	737,318(45,178)	744,115(92,304)	0.7^b^
NGM mm^3^	932,671(179,778)	801,051(53,533)	1,070,872(160,836)	<0.001^b^
Change NBV %/year	−0.06(0.66)	−0.035(0.52)	−0.09(0.78)	0.7^b^
Change NWM %/year	0.15(0.57)	0.021 (0.504)	0.29(0.60)	<0.031^b^
Change NGM %/year	0.02(0.75)	0.006 (0.883)	0.03(0.60)	0.9^b^
aPBVC	0.14(1.13)	0.03(0.88)	0.25(1.34)	0.4^b^
**Follow-Up**				
Days of Follow-Up	1,084(245)	1,178(262)	985(181)	<0.001^b^
EDSS	1.93(1.27)	1.48(1.07)	2.41(1.29)	0.001^b^
Delta EDSS	0.32(0.97)	0.37(0.92)	0.26(1.03)	0.6^b^
EDSS better *n*(%)	7 (9)	3 (7)	4 (10)	1.0^a^
EDSS stable *n*(%)	55(67)	28 (67)	27 (68)	1.0^a^
EDSS worse *n*(%)	20 (24)	11 (26)	9 (23)	1.0^a^
Treatment *n*(%)			0.2^a^
De-escalation	10(12)	Not applicable	10(25)	
No change	59(72)	35(83)	24(60)	
Escalation	13(16)	7(17)	6(15)	

**Notes.**

Data presented as mean (sd) if not indicated otherwise.

TITLE Delta absolute differences Change relative difference per month EDSS Expanded Disability Status Scale T2-Lesion hyperintense on T2 weighted images T1-Lesion hypointense on T1 weighted images GD-lesions Contrast enhancing lesions NBV normalized brain volume NGV normalized grey matter volume NWM normalized white matter volume

Volumes only from SIENAX.

a Differences between treated and untreated patients were tested with Chi-square test.

b *t*-tests.

c Mann–Whitney-*U*-test for ordinal data.

## Results

### Cohort

From 109 patients recruited, a clinical follow-up of at least 1.5 years was available with 82 (75%) patients. Mean follow-up time was three years (range 1.5–4.2). Patients that did not attend the follow-up were younger (*p* = 0.018) and had shorter disease duration at baseline (*p* < 0.001) than the follow-up cohort but did not differ in any other baseline parameters. In the follow-up cohort, 40 patients received DMDs and 42 were without medication. Six patients were treated with glatirameracetate, 19 with INF and 15 with natalizumab. About a third of patients had an EDSS progression at last follow-up. Descriptive statistics of the follow-up cohort are summarized in Table 1.

The variable time span between the baseline visits (median 7 months, range 5–14) was not associated with progression nor were treatment status (stable or changed, Chi square *p* = 0.2, ANOVA *p* = 0.1) or escalation/de-escalation (Chi square *p* = 0.2, ANOVA *p* = 0.3). Further analyses were not corrected for these potential confounders. Treated and untreated patients differed in follow-up time, EDSS and NBV at baseline (all *p* < 0.001) and we adjusted further analyses for treatment status.

### Validation of predefined classification algorithm

None of the predefined classification algorithms in high/low inflammation and atrophy groups were able to predict EDSS progression (Table 2)—except for one: change of FreeSurfer brain volume (Criterion 3) and at least four T2-lesions per year (Criterion C) in the whole cohort (*p* = 0.037). However, the algorithm failed to predict absolute EDSS change if adjusted for treatment status (*p* = 0.261) and comparison of the three different atrophy measurements was not possible.

**Table 2:** Predefined classification algorithms.

Inflammation criteri	Atrophy criteria	High inflammation and low atrophy	High inflammation and low atrophy	Low inflammation and high atrophy	High inflammation and high atrophy	Chi-square	Anova
A	1	33	18	10	21	0.344	0.382
	2	24	27	16	15	0.214	0.521
	3	32	19	15	16	0.184	0.262
	4	24	27	15	16	0.877	0.477
B	1	35	18	8	21	0.304	0.806
	2	26	27	14	15	0.093	0.529
	3	34	19	13	16	0.195	0.469
	4	26	27	13	16	0.768	0.861
C	1	40	23	3	16	0.152	0.189
	2	29	34	11	8	0.089	0.614
	3	38	25	9	10	0.037^*^	0.261
	4	29	34	10	9	0.470	0.545
D	1	33	18	10	21	0.344	0.382
	2	24	27	16	15	0.214	0.521
	3	32	19	15	16	0.184	0.262
	4	24	27	15	16	0.877	0.477

**Notes.**

Predictive value of predefined classification algorithms allocating patients based on two MRI five to 14 months apart in high/low inflammatory and atrophy groups. ANOVA adjusted for treatment status. For details see method section.

* *p* < 0.05.

### Explorative classification algorithms

**Figure 1:**
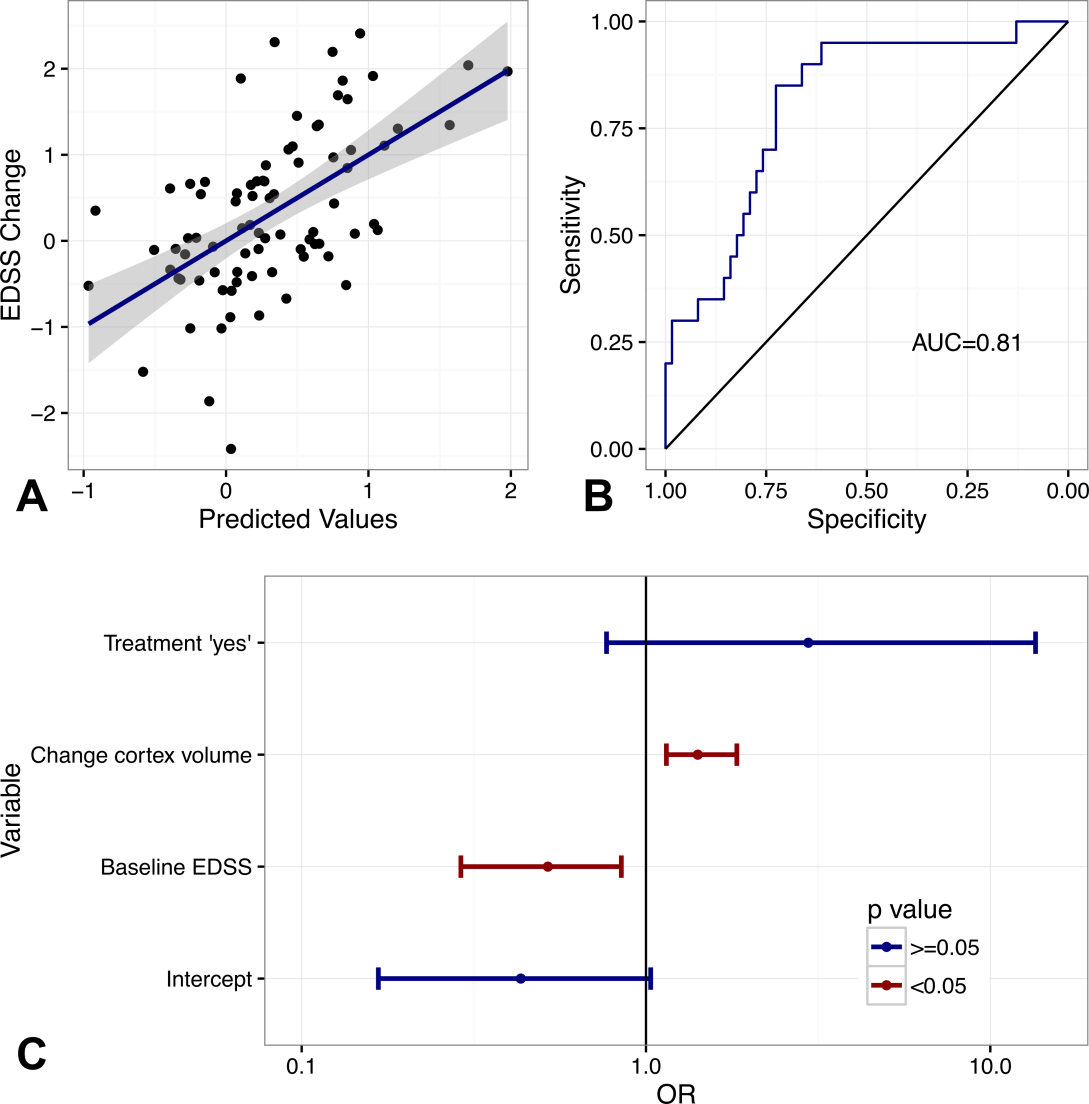
Predictive value of multivariate models. (A) Predicted values (multivariate linear model) and EDSS change. Dotplots and regression estimate (blue line) including 95%-CI (grey area). (B) ROC-curves of multivariate logit model predicting EDSS progression. (C) Odds ratios (OR) and 95% CI, red, significant, blue not significant. See Table 4 as well.

The results of screening predictors are summarised in Table 3. SIENA and SIENAX measurements were not significantly associated. Corrected for multiple testing only baseline EDSS, change of total grey matter volume and change of cortical grey matter remained significant. After stepwise selection of variables, the final multivariate linear model included treatment status, baseline EDSS and change of FreeSurfer grey matter volume (Table 4 and Fig. 1) as predictors (*R*^2^: 0.29). The corresponding logit model included cortical grey matter instead of total grey matter (Table 4). Separation between patients with and without progression was good (AUC = 0.81, Table 4 and Fig. 1). While higher atrophy indicated a higher risk of progression in all models, the association of baseline EDSS and progression was inverse, i.e., patients with lower EDSS had a higher risk to progress.

**Table 3:** Predictors of EDSS progression.

	Linear model		Logit models	
	*p*-value	FDR corrected *p*-value	*p*-value	FDR corrected *p*-value
Gender	0.048^*^	0.144	0.309	0.467
Age	0.156	0.312	0.337	0.467
Baseline EDSS	<0.001^*^	<0.001^*^	0.010*	0.072
Baseline T2-lesions	0.230	0.436	0.317	0.467
Baseline T1-lesions	0.273	0.467	0.242	0.436
Baseline Gd-lesions	0.453	0.604	0.868	0.913
Delta T2-lesions	0.586	0.681	0.038^*^	0.137
Delta T1-lesions	0.815	0.889	0.509	0.611
Change NBV	0.497	0.611	0.092	0.220
Change NWM	0.335	0.467	0.071	0.185
Change NGM	0.484	0.611	0.045^*^	0.145
aPBVC	0.974	0.974	0.707	0.795
Change cortex volume	0.020^*^	0.090	0.002^*^	0.036^*^
Change white matter volume	0.888	0.913	0.318	0.467
Change subcortical grey matter volume	0.101	0.220	0.026^*^	0.104
Change total grey mater volume	0.014^*^	0.072	0.004^*^	0.048^*^
Change supratentorial brain volume	0.104	0.220	0.009^*^	0.072
Change total brain volume	0.074	0.185	0.012^*^	0.072

**Notes.**

* *p* < 0.05.

**Table 4:** Multivariate models and EDSS progression.

	**Linear model**		
	*R*^2^ = 0.29		
Variables	Coeff	se	*p*-value
Intercept	0.723	0.149	<0.001
Treatment “yes”	0.433	0.202	0.035
Baseline EDSS	−0.370	0.071	<0.001
Change total grey matter volume	0.129	0.419	0.003
	**Logit model**		
Area under the curve	0.82		
Specificity %	72.6		
Sensitivity %	85.0		
NPV %	93.8		
PPV %	50.0		
**Variables**	**OR**	**95% CI**	*p*-**value**
Intercept	0.43	0.17–1.03	0.068
Treatment “yes”	2.96	0.77–13.54	0.132
Baseline EDSS	0.52	0.29–0.85	0.004
Change cortex volume	0.71	0.55–0.87	0.016

**Notes.**

Multivariate models investigating the predictive value of clinical and MRI measurements for EDSS progression. Details see methods and results.

Data presented as mean (sd) if not indicated otherwise.

TITLE Coeff coefficient estimate se standard error of Coeff NPV Negative Predictive Value PPV Positive Predictive Value OR Odds ratio 95% CI 95% Confidence Interval

## Discussion

So far only lesion load and new lesions (within restrictions) can be used as individual predictors of disease progression in routine imaging of MS patients. We identified short-term grey matter atrophy as a potential better predictor. Except from a low predictive value of Gd-enhancing lesions in treated patients, no lesion measurement was related to progression. From a pathophysiological perspective, it is feasible to combine measurements of inflammation and neurodegeneration to predict disability accumulation after several years (Bielekova et al., 2005). Here, all but one simple classification algorithms of high and low inflammatory or atrophy groups failed to foresee EDSS progression (Bielekova et al., 2005; Fisniku et al., 2008; Popescu et al., 2013; Jacobsen et al., 2014; Tintore et al., 2015).

The negligible sensitivity of lesions in our cohort might be explained by the fact that previous studies mainly investigated patients with a clinically isolated syndrome (CIS) while we investigated established RRMS (Fernández, 2013; Odenthal & Coulthard, 2015; Tintore et al., 2015). It is well known from natural history data that relapses do not influence the risk of disability or the onset of a progressive disease course if they occur later than two years after disease onset (Degenhardt et al., 2009; Scalfari et al., 2010). We assume that our patients were in a later phase of the disease where T2-lesions may have only a minor impact which is in accordance with other cohorts (Jacobsen et al., 2014; Uher et al., 2015) but not all (Popescu et al., 2013). The association between lesions and relapses could not be evaluated as information about relapses was not reliable, but based on the considerations stated this is not a major limitation.

In our cohort, grey matter atrophy was more predictive than total brain or white matter atrophy. This observation is in line with previous studies, where progression was associated with cortical atrophy and subcortical grey matter changes (Rocca et al., 2013; Jacobsen et al., 2014) It is known that clinical disability is closer associated with cortical pathology than with T2-lesions or normal appearing white matter (Filippi et al., 2013). DMDs or their change were not associated with disability progression. The missing effect of immunotherapies might be due to inconsistent treatment effects of DMD. Again, these findings are in line with previous studies (Daumer et al., 2009; Hauser, Chan & Oksenberg, 2013). So far, most of the reported associations between grey matter and disability are based on absolute volumes e.g., grey matter volume from a single MRI (Rocca et al., 2013; Jacobsen et al., 2014; Tintore et al., 2015). The use of absolute cut-offs as predictors, for example, Bielekovas’ 83% brain parenchymal fraction (Bielekova et al., 2005) are restricted and specific to each cohort as different scanners, and sequences and processing pipelines have a major influence on these values and calibration is not possible (Obuchowski et al., 2014). For example, even in our cohort the absolute brain volumes differed between treated and untreated patients and inversely as assumed; treated and more disabled patients with longer disease duration had higher baseline brain volume. As the scanner sequences and analysis pipeline were the same the observation must be due to an unknown bias. Using relative values such as changes from baseline, is a feasible approach to overcome such short-comings even though they are less informative than calibrated quantitative measurements (Obuchowski et al., 2014). In our study we used three different kinds of relative values (Fischl, 2012). Only the FreeSurfer algorithm was associated with progression and seems to be more reliable and sensitive than SIENA/SIENAX. However, computing these measurements still requires several hours and is not yet feasible for clinical routine.

The higher risk for progression in patients with lower EDSS seems counterintuitive at first sight. EDSS scores below four represent mainly the neurological examination (Kurtzke, 1983) and even non-disabling new symptoms may lead to an increase of the EDSS. Most of our patients had no or only mild disability at baseline. Therefore EDSS-progression represents non-disabling symptoms in most cases. Whether or not such EDSS changes are predictive in the long-run is questionable and cannot (could not?) be improved by confirming EDSS changes after three or six months which was not possible in our cohort (Ebers et al., 2008). Over all, the risk of progression in our cohort was in line with other cohorts (Jacobsen et al., 2014). Furthermore the median time from disease onset to EDSS three is about 12 years which is still above the median disease duration at follow-up in our cohort (Scalfari et al., 2010). Our findings are somehow limited as 25% were lost to follow-up, which is similar to other cohorts (Jacobsen et al., 2014). Accounting for heterogeneous follow-up by implementing survival analyses was not possible as the lack of independency of censoring violates a fundamental assumption of survival analyses (Leung, Elashoff & Afifi, 1997). As dropouts did not differ relevantly from follow-up patients, we assume no major impact on our results. Our relatively small sample size restricts the generalization of our findings but overall FreeSurfer measurements are a promising method to enhance individual risk stratification.

## Conclusion

Besides EDSS at baseline, grey matter atrophy within one year is a valuable predictor for EDSS progression in treated and untreated RRMS. The development of atrophy measurements for individual risk counselling and evaluation of treatment response seems possible but defining a simple to compute generalizable measurement is still challenging.

##  Supplemental Information

10.7717/peerj.2442/supp-1 Data S1 CSV table containing demographic, EDSS and MRI data for each patient

## Additional Information and Declarations

### Data Availability

The following information was supplied regarding data availability: The raw data has been supplied as Data S1.

## References

[ref-1] Benedict RHB, Carone DA, Bakshi R (2004). Correlating brain atrophy with cognitive dysfunction, mood disturbances, and personality disorder in multiple sclerosis. Journal of Neuroimaging.

[ref-2] Bielekova B, Kadom N, Fisher E, Jeffries N, Ohayon J, Richert N, Howard T, Bash CN (2005). MRI as a marker for disease heterogeneity in multiple sclerosis. Neurology.

[ref-3] Daumer M, Neuhaus A, Morrissey S, Hintzen R, Ebers GC (2009). MRI as an outcome in multiple sclerosis clinical trials. Neurology.

[ref-4] Degenhardt A, Ramagopalan SV, Scalfari A, Ebers GC (2009). Clinical prognostic factors in multiple sclerosis: a natural history review. Nature Reviews Neurology.

[ref-5] Ebers GC, Heigenhauser L, Daumer M, Lederer C, Noseworthy JH (2008). Disability as an outcome in MS clinical trials. Neurology.

[ref-6] Fernández O (2013). Integrating the tools for an individualized prognosis in multiple sclerosis. Journal of the Neurological Sciences.

[ref-7] Filippi M, Rocca MA, Horsfield MA, Hametner S, Geurts JJG, Comi G, Lassmann H (2013). Imaging cortical damage and dysfunction in multiple sclerosis. JAMA Neurology.

[ref-8] Fischl B (2012). Free surfer. NeuroImage.

[ref-9] Fisniku LK, Brex PA, Altmann DR, Miszkiel KA, Benton CE, Lanyon R, Thompson AJ, Miller DH (2008). Disability and T2 MRI lesions: a 20-year follow-up of patients with relapse onset of multiple sclerosis. Brain.

[ref-10] Friese MA, Schattling B, Fugger L (2014). Mechanisms of neurodegeneration and axonal dysfunction in multiple sclerosis. Nature Reviews Neurology.

[ref-11] Hauser SL, Chan JR, Oksenberg JR (2013). Multiple sclerosis: prospects and promise. Annals of Neurology.

[ref-12] Jacobsen C, Hagemeier J, Myhr K-M, Nyland H, Lode K, Bergsland N, Ramasamy DP, Dalaker TO, Larsen JP, Farbu E, Zivadinov R (2014). Brain atrophy and disability progression in multiple sclerosis patients: a 10-year follow-up study. Journal of Neurology, Neurosurgery, and Psychiatry.

[ref-13] Kurtzke JF (1983). Rating neurologic impairment in multiple sclerosis: an expanded disability status scale (EDSS). Neurology.

[ref-14] Leung KM, Elashoff RM, Afifi A (1997). Censoring issues in survival analysis. Annual Review of Public Health.

[ref-15] Obuchowski NA, Reeves AP, Huang EP, Wang X-F, Buckler AJ, Kim HJG, Barnhart HX, Jackson EF, Giger ML, Pennello G, Toledano AY, Kalpathy-Cramer J, Apanasovich TV, Kinahan PE, Myers KJ, Goldgof DB, Barboriak DP, Gillies RJ, Schwartz LH, Sullivan ADC (2014). Quantitative imaging biomarkers: a review of statistical methods for computer algorithm comparisons. Statistic Methods in Medical Research.

[ref-16] Odenthal A, Coulthard C (2015). The prognostic utility of MRI in clinically isolated syndrome: a literature review. AJNR. American Journal of Neuroradiology.

[ref-17] Perez-Miralles FC, Sastre-Garriga J, Vidal-Jordana A, Rio J, Auger C, Pareto D, Tintore M, Rovira A, Montalban X (2015). Predictive value of early brain atrophy on response in patients treated with interferon. Neurology: Neuroimmunology & Neuroinflammation.

[ref-18] Polman CH, Reingold SC, Banwell B, Clanet M, Cohen JA, Filippi M, Fujihara K, Havrdova E, Hutchinson M, Kappos L, Lublin FD, Montalban X, O’Connor P, Sandberg-Wollheim M, Thompson AJ, Waubant E, Weinshenker B, Wolinsky JS (2011). Diagnostic criteria for multiple sclerosis: 2010 revisions to the “McDonald criteria”. Annals of Neurology.

[ref-19] Popescu V, Agosta F, Hulst HE, Sluimer IC, Knol DL, Sormani MP, Enzinger C, Ropele S, Alonso J, Sastre-Garriga J, Rovira A, Montalban X, Bodini B, Ciccarelli O, Khaleeli Z, Chard DT, Matthews L, Palace J, Giorgio A, De Stefano N, Eisele P, Gass A, Polman CH, Uitdehaag BMJ, Messina MJ, Comi G, Filippi M, Barkhof F, Vrenken H (2013). Brain atrophy and lesion load predict long term disability in multiple sclerosis. Journal of Neurology, Neurosurgery and Psychiatry.

[ref-20] Reuter M, Schmansky NJ, Rosas HD, Fischl B (2012). Within-subject template estimation for unbiased longitudinal image analysis. NeuroImage.

[ref-21] Rio J, Rovira A, Tintoré M, Huerga E, Nos C, Tellez N, Tur C, Comabella M, Montalban X (2008). Relationship between MRI lesion activity and response to IFN-beta in relapsing-remitting multiple sclerosis patients. Multiple Sclerosis.

[ref-22] Rocca M, Preziosa P, Copetti M, Riccitelli GC, Messina R, Comi G, Filippi M (2013). Gray matter damage predicts the accumulation of disability and cognitive impairment 13 years later in MS. Neurology.

[ref-23] Scalfari A, Neuhaus A, Degenhardt A, Rice GP (2010). The natural history of multiple sclerosis: A geographically based study 10: relapses and long-term disability. Brain: a Journal of Neurology.

[ref-24] Smith SM, Jenkinson M, Woolrich MW, Beckmann CF, Behrens TEJ, Johansen-berg H, Bannister PR, Luca MD, Drobnjak I, Flitney DE, Niazy RK, Saunders J, Vickers J, Zhang Y, De Stefano N, Brady JM, Matthews PM (2004). Advances in functional and structural MR image analysis and implementation as FSL. Neuroimage.

[ref-25] Smith SM, Zhang Y, Jenkinson M, Chen J, Matthews PM, Federico A, De Stefano N (2002). Accurate, robust, and automated longitudinal and cross-sectional brain change analysis. NeuroImage.

[ref-26] Sormani MP, Bonzano L, Roccatagliata L, Cutter GR, Mancardi GL, Bruzzi P (2009). Magnetic resonance imaging as a potential surrogate for relapses in multiple sclerosis: a meta-analytic approach. Annals of Neurology.

[ref-27] Sormani MP, De Stefano N (2013). Defining and scoring response to IFN-beta in multiple sclerosis. Nature Reviews Neurology.

[ref-28] Steenwijk MD, Geurts J, Daams M, Tijms BM, Wink AM, Balk LJ, Tewarie PK, Uitdehaag BMJ, Barkhof F, Vrenken H, Pouwels PJW (2016). Cortical atrophy patterns in multiple sclerosis are non-random and clinically relevant. Brain.

[ref-29] Stellmann J-P, Stürner KH, Young KL, Siemonsen S, Friede T, Heesen C (2015). Regression to the mean and predictors of MRI disease activity in RRMS Placebo cohorts—is there a place for baseline-to-treatment studies in MS?. PLoS ONE.

[ref-30] Tintore M, Rovira A, Rio J, Otero-Romero S, Arrambide G, Tur C, Comabella M, Nos C, Arevalo MJ, Negrotto L, Galan I, Vidal-Jordana A, Castillo J, Palavra F, Simon E, Mitjana R, Auger C, Sastre-Garriga J, Montalban X (2015). Defining high, medium and low impact prognostic factors for developing multiple sclerosis. Brain.

[ref-31] Uher T, Horakova D, Kalincik T, Bergsland N, Tyblova M, Ramasamy DP, Seidl Z, Vaneckova M, Krasensky J, Havrdova E, Zivadinov R (2015). Early magnetic resonance imaging predictors of clinical progression after 48 months in clinically isolated syndrome patients treated with intramuscular interferon *β*-1a. European Journal of Neurology.

[ref-32] Zivadinov R, Uher T, Hagemeier J, Vaneckova M, Ramasamy DP, Tyblova M, Bergsland N, Seidl Z, Dwyer MG, Krasensky J, Havrdova E, Horakova D (2016). A serial 10-year follow-up study of brain atrophy and disability progression in RRM Spatients. Multiple Sclerosis Journal.

